# A pathogen protease‐activated molecular decoy for customized resistance in plant

**DOI:** 10.1111/pbi.70016

**Published:** 2025-03-26

**Authors:** Xinyue Fan, Yu Zhao, Weiqin Ji, Bernardo Rodamilans, Carmen Simón‐Mateo, Juan Antonio García, Xiaoxia Wu, Xiaoyun Wu, Xiaofei Cheng

**Affiliations:** ^1^ College of Plant Protection Northeast Agricultural University Harbin Heilongjiang China; ^2^ Department of Plant Molecular Genetics, Centro Nacional de Biotecnología‐CSIC Campus Universidad Autónoma de Madrid Madrid Spain; ^3^ College of Agriculture Northeast Agricultural University Harbin Heilongjiang China

**Keywords:** customized resistance, protease‐activated molecular decoy, plant elicitor peptides

Breeding for disease‐resistant crops is essential to ensure global food security. Traditional crop breeding, while widely accepted, is time‐consuming and is always hampered by the scarcity of resistance sources. Although customizing disease resistance has been a long‐cherished dream for breeders, data for a few successful examples are available. Recent advances in plant immune mechanisms have made it possible to genetically engineer the effector binding interface of intracellular nucleotide‐binding leucine‐rich repeat (NLR) proteins to create artificial resistance (Contreras *et al*., 2023; Kim *et al*., 2016). However, NLR requires complex structural changes for activation; maladaptive changes can result in a non‐functioning or trailing necrotic phenotype (Pottinger *et al*., 2020).

Plant elicitor peptides (Peps) are peptide damage‐associated molecular patterns (DAMPs) of approximately 23 amino acids (aa) in length that are widely distributed throughout the plant kingdom (Yamaguchi and Huffaker, 2011). Under homeostatic conditions, Peps are present in cells in the form of precursors (proPeps) but are rapidly released from the C‐terminus of proPeps by type‐II metacaspases (MCs) in response to cellular damage caused by injury or infection (Shen *et al*., 2019). Mature forms of Peps are then released into the apoplasm, where they are recognized by cell surface‐localized pattern recognition receptors (PRRs), initiating resistance responses such as the expression of defence genes, deposition of callose and synthesis of phytohormones (Hou *et al*., 2019). Pep‐induced immunity confers a broad‐spectrum resistance to various phytopathogens.

We hypothesized that proPep could be engineered as a molecular decoy by replacing the MC cleavage site with the cleavage sites of other pathogen proteases. As a result, the modified ProPep is hydrolysed by the target pathogen's protease to induce an immune response. To test this hypothesis, we amplified the full‐length transcript of proPep1, one of the eight proPep paralogs of *Arabidopsis thaliana*. A previous study suggested that proPep1 is hydrolysed by type‐II MCs after the Arg residue at position 69 (Shen *et al*., 2019). We therefore replaced the amino acids between aa 66–69 (VTSR↓AT) with the cleavage site (VYHQ↓A) of the major protease (NIa‐Pro) of turnip mosaic virus (TuMV) (Kang *et al*., 2001), a member of the genus *Potyvirus* in the family *Potyviridae* (Figure 1a). We transiently expressed this modified proPep1 as a C‐terminal YFP‐tagged recombinant protein in *Nicotiana benthamiana* leaves under the CaMV 35S promoter, alone or together with the N‐terminal FLAG‐4×Myc‐tagged TuMV NIa‐Pro, a non‐functional mutant of this protease (NIa‐Pro^C151A^), or TuMV‐6K2mCherry, a TuMV infectious clone containing an additional mCherry‐tagged 6K2 between P1 and HcPro cistrons (Cotton *et al*., 2009). At 48 h post‐infiltration (hpi), the leaves were harvested and analysed by Western blotting with antibodies against GFP and Myc, respectively. The results showed that a single band of about 40 kDa was detected in the sample expressing proPep1^NIa‐Pro^‐YFP alone or co‐expressed with NIa‐Pro^C151A^, whereas an additional band of about 30 kDa was detected when co‐expressed with NIa‐Pro or TuMV‐6k2mCherry (Figure 1b), indicating that proPep1^NIa‐Pro^ was successfully hydrolysed by transiently expressed NIa‐Pro or NIa‐Pro produced during viral infection. In addition, the plasmolysis assay revealed that YFP foci would be observed in the apoplast space in TuMV‐infested cells, whereas no YFP signal was observed in the apoplast space of control plants (Figure S1), suggesting that mature Pep1^NIa‐Pro^‐YFP can be secreted into the apoplast space.

**Figure 1:**
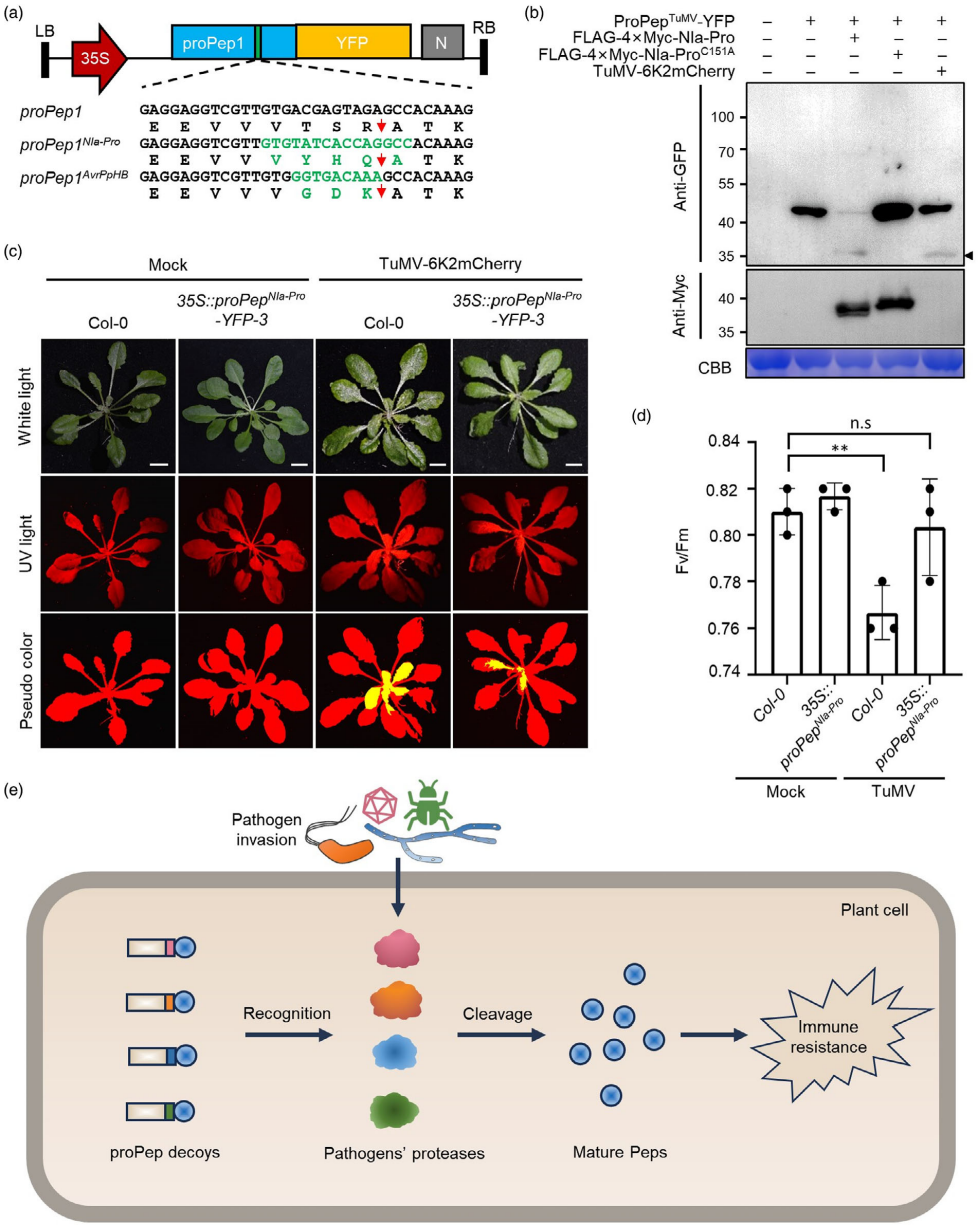
Engineered elicitor peptide precursor confers resistance. (a) Illustration of constructs expressing YFP‐tagged proPep1 and its mutants carrying the recognition hexapeptides of TuMV NIa‐Pro (proPep1^NIa‐Pro^) or *Pseudomonas syringae* PpHB (proPep1^AvrPpHB^). LB, T‐DNA left border; RB, T‐DNA right border; 35S, cauliflower mosaic virus 35S promoter; N, NOS terminator; red arrows indicate the cleavage sites. (b) Western blotting for the cleavage of transiently expressed proPep1^NIa‐Pro^‐YFP by FLAG‐4×Myc‐NIa‐Pro, FLAG‐4×Myc‐NIa‐Pro^C151A^, or TuMV‐6K2mCherry in *N. benthamiana* leaves at 2 dpi. The black arrowhead indicates the cleavage product. (c) Phenotype of mock‐ or TuMV‐6K2mCherry‐infected WT and transgenic plants under white and UV light at 11 dpi. Red and yellow colours in the pseudocolour panel refer to virus‐free and ‐infected areas, respectively. (d) Bar graph of the Fv/Fm of WT and transgenic plants at 11 dpi (*n* = 3). (e) Schematic model of the pathogen protease‐activated resistance system.

To test whether this modified proPep1^NIa‐Pro^ could confer resistance to TuMV, we generated transgenic *A. thaliana* expressing proPep1^NIa‐Pro^‐YFP under the CaMV 35S promoter (*35S::proPep1* ^*NIa‐Pro*^ *‐YFP*). All homozygous transgenic seedlings had phenotypes similar to WT plants (Figure S2), indicating that overexpression of prePep1^NIa‐Pro^ has no obvious effect on plant growth under steady‐state conditions. We then inoculated 4‐week‐old seedlings of wild‐type Col‐0 (WT) and *35S::proPep1* ^*NIa‐Pro*^ *‐YFP‐3* with TuMV‐6K2mCherry by agroinfiltration. At 11 days post‐inoculation (dpi), we found that the TuMV‐infected leaf area of transgenic seedlings was significantly smaller than that of WT seedlings, as indicated by the fluorescence (Figure 1c; Figure S3a). Western blotting confirmed that proPep1^NIa‐Pro^‐YFP was successfully hydrolysed by TuMV‐6K2mCherry‐encoded NIa‐Pro (Figure S3b). RT‐qPCR with total RNA extracted from total aerial tissues showed that transgenic seedlings accumulated only about half as much viral genomic RNA compared to that in TuMV‐infected WT plants (Figure S3c). In addition, RT‐qPCR showed that the expression of *R1* was also significantly higher in TuMV‐infected transgenic seedlings compared with that in TuMV‐infected WT plants (Figure S3d). We further assessed the stress of these plants using the Fv/Fm (variable fluorescence/maximum fluorescence) assay (Chauhan *et al*., 2023). The Fv/Fm ratio represents an estimate of the maximum photochemical efficiency of PSII, and a decrease in this ratio indicates the closure of reaction centres caused by biotic or abiotic stress (Chauhan *et al*., 2023). The results showed that TuMV‐infected transgenic plants had a similar Fv/Fm ratio to mock control plants, whereas TuMV‐infected WT plants had a significantly reduced Fv/Fm ratio (Figure 1d), indicating that TuMV infection induced significant cellular stress, which was greatly reduced in the transgenic plants. To exclude the possibility that the resistance was not due to Agrobacterium stimuli, we inoculated WT and two transgenic lines with TuMV‐6K2mCherry by mechanical inoculation. The results showed that both transgenic lines exhibited enhanced resistance to TuMV‐6K2mCherry (Figure S4). We also agroinoculated WT and *35S::proPep1* ^*NIa‐Pro*^ *‐YFP‐3* seedlings with beet severe curly top virus (BSCTV), a DNA virus of the family *Geminiviridae*. The results showed that there was no significant difference in BSCTV infectivity between transgenic and WT plants (Figure S5), confirming that the resistance is specific to TuMV. Taken together, these data demonstrate that Pep1 can be modified to confer resistance to TuMV with a negligible growth trade‐off.

To further confirm the suitability of proPep1 for engineering as a molecular decoy, we replaced the MC cleavage site in proPep1 with the cleavage site of AvrPphB (GDK↓X; X represents any amino acid) to construct proPep1^AvrPphB^ (Figure 1a). AvrPphB is a cysteine protease from *Pseudomonas syringae* that can cleave a small family of receptor‐like cytoplasmic kinases, including PBS1, which acts as a ‘decoy’ to activate the RPS5‐mediated HR response upon cleavage by AvrPphB. We transiently expressed YFP‐tagged proPep1^AvrPphB^ alone or together with mRFP‐tagged AvrPphB or its dysfunctional mutant (AvrPphB^C98A^) under the CaMV 35S promoter in the leaves of *N. benthamiana*. Immunoprecipitation followed by Western blot analysis showed that proPep1^AvrPphB^‐YFP was readily cleaved by AvrPphB but not by AvrPphB^C98A^ (Figure S6a), indicating that proPep1 can also be engineered to be recognized by a bacterial protease. We also generated transgenic plants expressing proPep1^AvrPphB^‐YFP under the CaMV 35S promoter in the *rps5* background (*rps5 35S::proPep1* ^*AvrPphB*^ *‐YFP*) to avoid the recognition of AvrPphB by RPS5. Transgenic seedlings expressing proPep1^NIa‐Pro^‐YFP had phenotypes similar to *rps5* plants (Figure S6b). We then inoculated 4‐week‐old seedlings of *rps5* and *rps5 35S::proPep1* ^*NIa‐Pro*^ *‐YFP‐3* with *P. syringae* pv. tomato DC3000 (*pst* DC3000) and assessed bacterial growth at 40 hpi. The results showed that the transgenic plants accumulated significantly lower levels of *pst* DC3000 than *rps5* seedlings (Figure S6c), suggesting that overexpression of proPep1^AvrPphB^ can also enhance the resistance to the corresponding bacterium.

Proteases are encoded by many phytopathogens and play a critical role in their pathogenicity (López‐Otín and Bond, 2008). Thus, this novel artificial disease resistance system can be easily adapted to other pathogens by simply replacing the cleavage site within the defense elicitor with that of a pathogen‐derived protease and can be pyramided against multiple pathogens for customized resistance (Figure 1e).

## Conflict of interest

The authors declare that they have no conflict of interest.

## Author contributions

X.C. and X.W. contributed to the project design. X.F., X.W., W.J. and Y.Z. performed the experiments and dataanalysis. B.R. and C.S.‐M. provided data analyses support. X.C. and X.W. wrote the manuscript. X.C. and J.A.G. revised the article. X.C. provided project supervision.

## Supporting information


**Figures S1-S6** Supplementary Figures.

**Table S1** Primers used in the research.

## Data Availability

The data that supports the findings of this study are available in the supplementary material of this article.
