# Supplementary material for: A pathogen protease‐activated molecular decoy for customized resistance in plant
Source: Plant Biotechnol J. 2025 Mar 26;23(6):2403–5. doi: 10.1111/pbi.70016 (PMC12120908; doi:10.1111/pbi.70016)
Supplement: Supplementary file 1 — Figures S1-S6 Supplementary Figures. [file PBI-23-2403-s002.pdf]

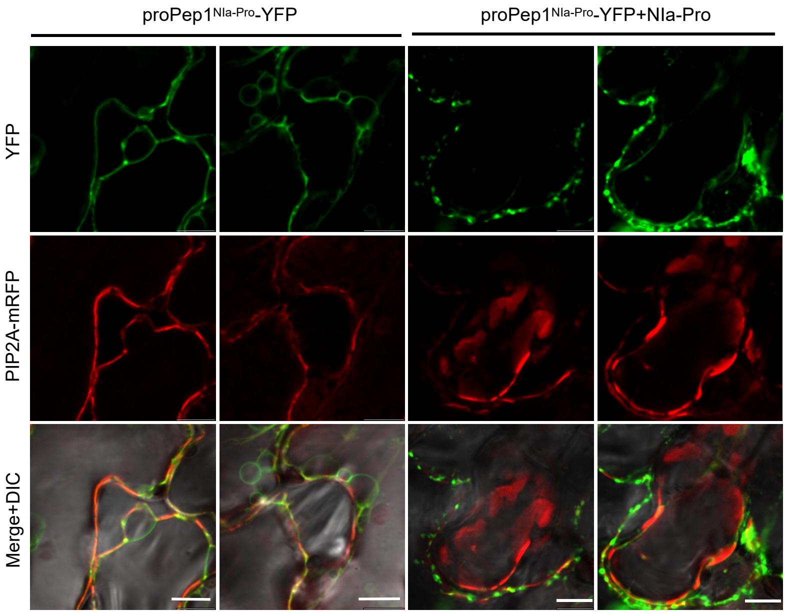

Supplemental Fig. 1

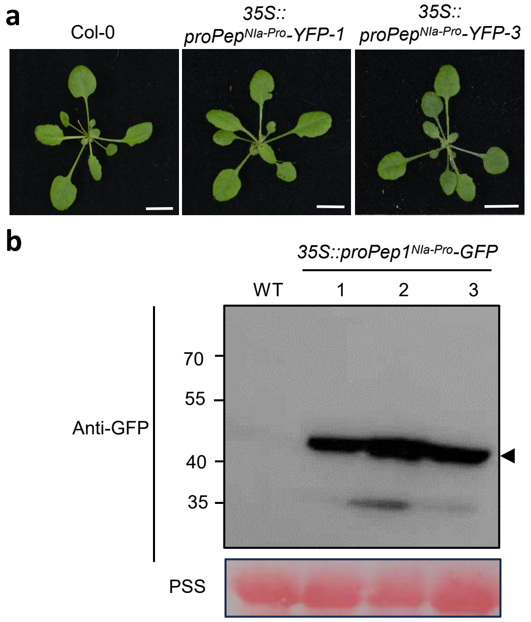

Supplemental Fig. 2

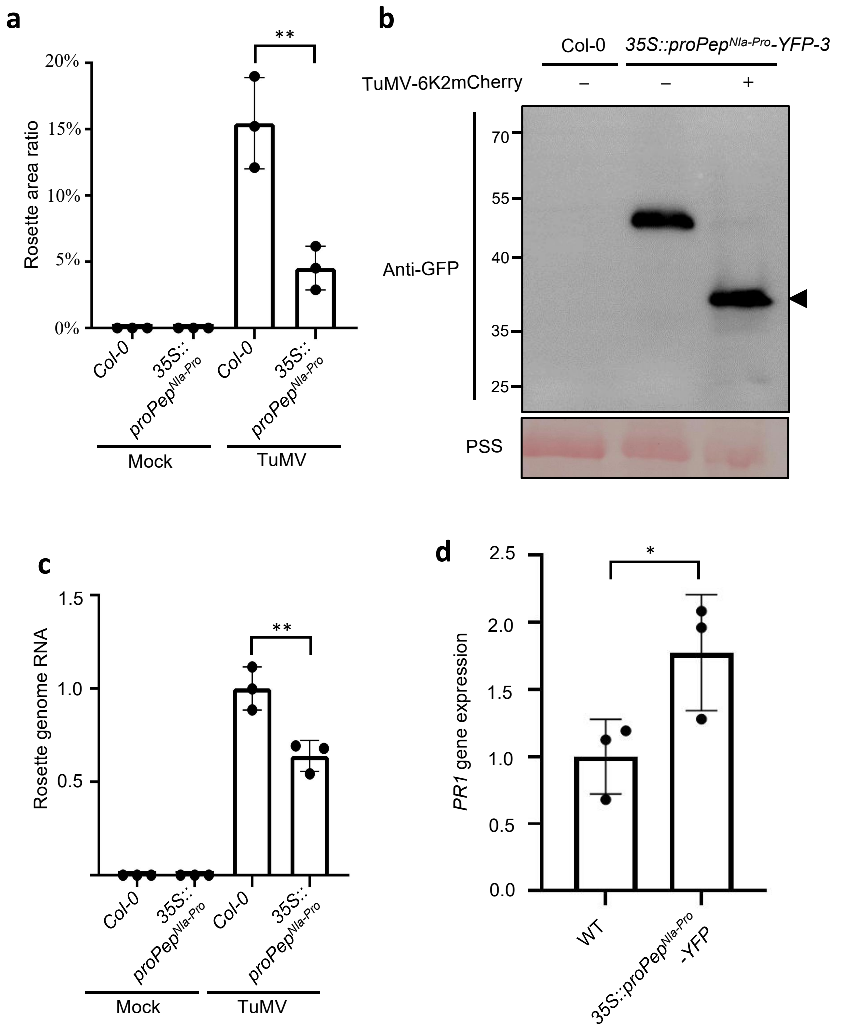

Supplemental Fig. 3

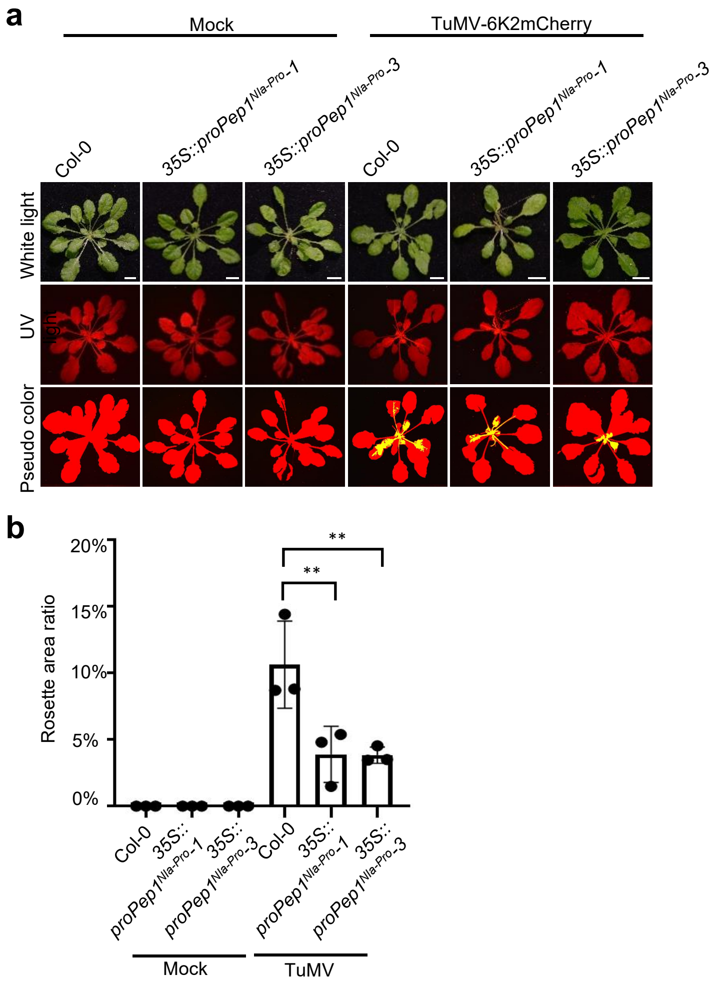

Supplemental Fig. 4

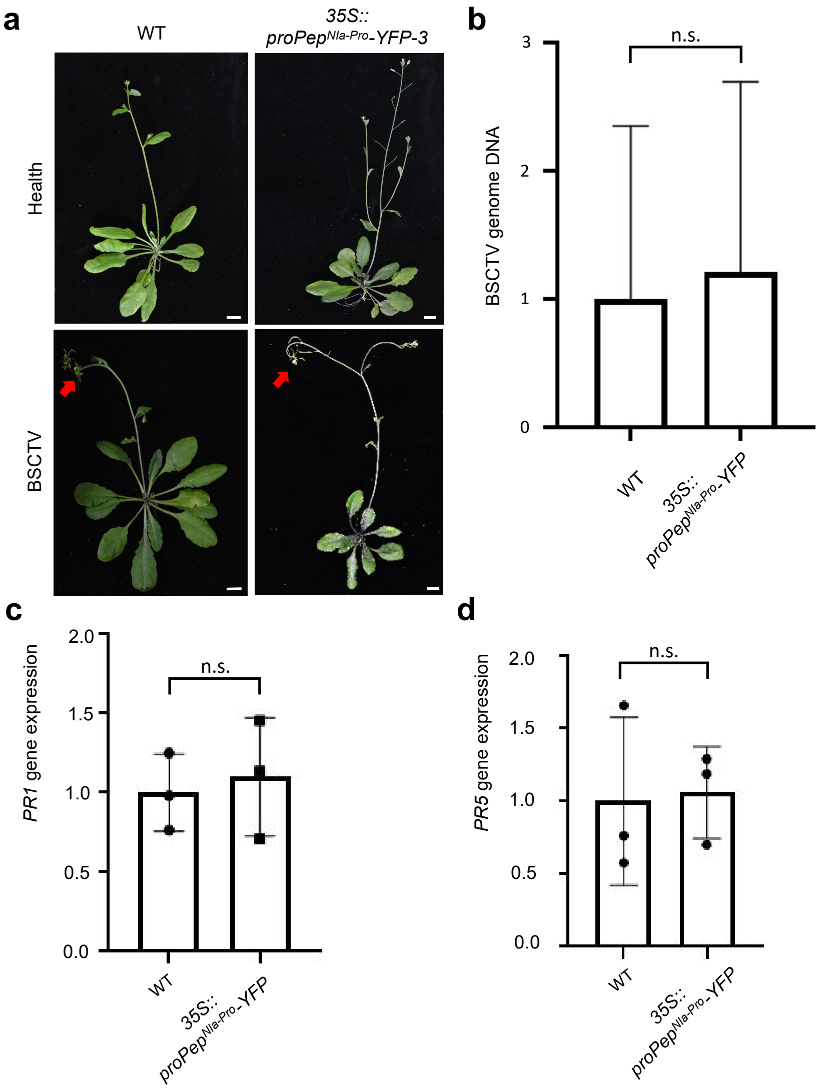

Supplemental Fig. 5

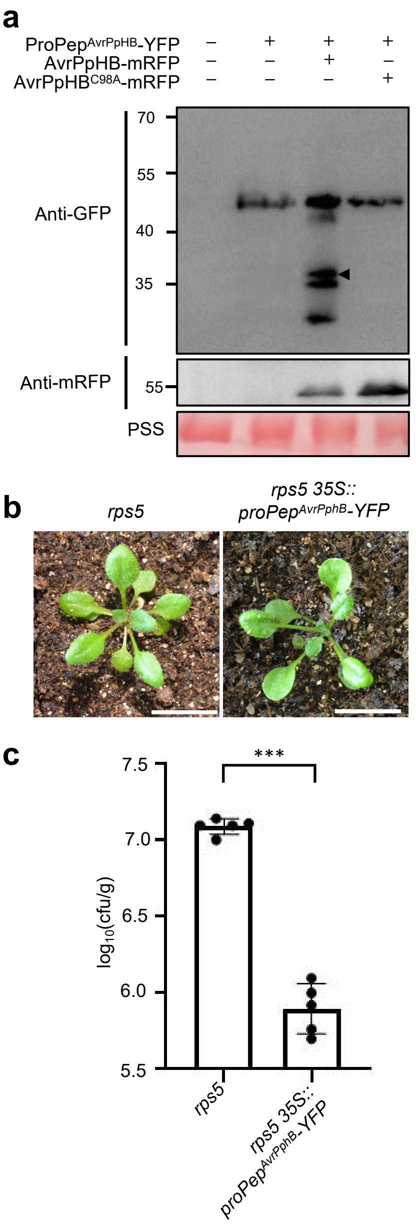

Supplemental Fig. 6
