# Supplementary material for: A pathogen protease‐activated molecular decoy for customized resistance in plant
Source: Plant Biotechnol J. 2025 Mar 26;23(6):2403–5. doi: 10.1111/pbi.70016 (PMC12120908; doi:10.1111/pbi.70016)
Supplement: Supplementary file 2 — Table S1 Primers used in the research. [file PBI-23-2403-s001.docx]

**Materials and Methods**

**Plant growth and transformation**

*N. benthamiana* and *Arabidopsis thaliana* seedlings were grown in pots in growth chambers at 23 °C with 16/8 (light/dark) and 12/12 photoperiods, respectively. Arabidopsis was transformed using the floral dip method as described previously([Bent, 2006](#_ENREF_1)).

**Agroinfiltration**

Plasmid was transformed into *Agrobacterium rhizogenes* strain GV3101 by electroporation. Agrobacteria were grown at 28 °C overnight in liquid Luria-Bertani medium containing suitable antibiotics, pelleted by centrifugation at 6000 rpm, resuspended in infiltration buffer (100 μM Acetosyringone; 10 mM MgCl_2_; 10 mM 2-Morpholinoethanesulfonic acid, pH 5.6), and then incubated at room temperature for about 2 h. The agrobacterial solution was infiltrated into the beaves of *N. benthamiana* or *A. thaliana* by a 1-mL needleless syringe at a final optical density at 600 nm (O.D._600_) of 0.4.

**Mechanical inoculation**

Mechanical inoculation was performed as described previously([Li et al., 2022](#_ENREF_5)). In brief, TuMV-6K2mCherry-infected *N. benthamiana* leaves were homologized in 10 mM phosphate buffer (pH 7.2) and the extract was directly used to rub *A. thaliana* leaves that predusted by 600-mesh carbo-rundum powder for 3–4 times. After air dry for 2–3 minutes, the inoculated leaves were washed by distilled water and covered with a wet paper towel. Plants were kept in a growth chamber for symptom development.

**Sampling**

For whole plant, the whole aerial part was ground in liquid nitrogen and used for subsequent total RNA extraction or immunoblotting. For transient expression, equal amount of leaf tissue was obtained using a biopsy punch and used for subsequent immunoblotting.

**Plasmid construction**

The full-length coding sequences of TuMV NIa-Pro (NC_002509) were amplified from the TuMV infectious DNA clone TuMV-GFP with primers listed in the Supplementary Table 1([Lellis et al., 2002](#_ENREF_4)). The coding sequence of proPep1 (NM_125888) was amplified from the cDNA reverse transcribed from healthy *A. thaliana*, while the coding sequence of AvrPpHB (M86401) was synthesized in GenScript Biotech Corporation (Nanjing, China). Mutations of proPep1, NIa-Pro and AvrPpHB were constructed by overlapping PCR with the primer listed in the Supplementary Table 1. All fragments were cloned into the Gateway-compatible vector pDONRR-207-SapI using the ClonExpress II (Vazyme Biotech Inc., Nanjing, China) and then transferred into the gateway-compatible destination vectors using the Gateway™ LR Clonase™ II Enzyme mix (Thermal Fisher Scientific, Shanghai, China). In detail, the pEarleyGate-101, pGWB-554, and pBA-FLAG-4×Myc-DC were used to construct C-terminal YFP-, mRFP, and N-terminal FLAG-4×Myc-tagged constructs, respectively ([Earley et al., 2006](#_ENREF_3), [Nakagawa et al., 2007](#_ENREF_6), [Zhu et al., 2011](#_ENREF_8)). All plasmids were verified by Sanger sequencing.

**Nucleic acid extraction, reverse transcription, and qPCR**

Total RNA was extracted using the Eastep® Super Total RNA Extraction Kit (Promega, Beijing, China). The first-strand of cDNA was synthesized using the HiScript® Ⅲ 1^st^ Strand cDNA Synthesis (+gDNA wiper) Kit (Vazyme). Quantitative PCR (qPCR) was performed in a 10 μl volume system with a CFX Connect^TM^ Real-Time System (Bio-Rad, California, USA). The genome RNA of TuMV was determined by amplification of a 257 bp fragment of the *CP* gene. The *A. thaliana* *EF1α* was used as the internal control. All primers are listed in Supplemental Table 1.

**Sodium dodecyl sulphate‒polyacrylamide gel electrophoresis (SDS‒PAGE) and Western blotting**

Approximately 0.1 g of plant tissue fine powder was mixed with 100 µL of 1 × SDS‒PAGE loading buffer (50 mM Tris HCl, pH 6.8; 10% glycerol; 2% SDS; 100 mM DTT; 0.005% bromophenol blue). The mixture was boiled at 95 °C for 5 min, clarified by centrifugation at 12,000 × g for 4 min at 4 °C, and then was directly used for SDS‒PAGE. Proteins were separated in 12% home-made polyacrylamide gels and transferred to polyvinylidene difluoride (PVDF) membrane using a Trans-Blot Turbo Transfer System. PVDF membrane was blocked in TBST containing 5% non-fat milk powder for 1 h at room temperature, was incubated with appropriate primary antibody (in TBST containing 5% non-fat milk) for additional 1 h at room temperature. After being washed in 8 times with TBST, the PVDF membrane was incubated with appropriate secondary antibody (in TBST containing 5% non-fat milk) for 1 h at room temperature. After being washed in 8 times in TBST, the PVDF membrane was visualized by a Tanon 5200CE Chemiluminescent Imaging System with the Immobilon Western Chemiluminescent HRP Substrate solution (Merch Millipore). A parallel gel was stained with the Coomassie Brilliant Blue as a loading control. Rabbit polyclonal anti-GFP N-terminal antibodies (Sigma‒Aldrich) and anti-Myc antibodies (Abcam) were used at 1:5000 and 1:2000 dilutions, respectively. HRP-conjugated anti-rabbit IgG secondary antibodies (Sigma-Aldrich) at a 1:10,000 dilution.

**Plasmolysis assay**

Plasmolysis was performed as previously described ([Cheng et al., 2017](#_ENREF_2)). In brief, an about 1×1 cm *N. benthamiana* leaf tissue was sliced off with a razor blade and placed in 30% sucrose solution for 30 min. The leaf tissue was then fixed to the slide and covered with a coverslip and observed using the Leica TCS SP8 Lightning laser confocal microscope.

**Chlorophyll fluorescence imaging**

Chlorophyll fluorescence of *Arabidopsis thaliana* was detected by FluorCam (Ecotech, Beijing, China). *A. thaliana* seedlings were dark-treated for 20 minutes before measure*.* The *F_0_* images were recorded in a quasi-dark state. The maximum fluorescence yield *F_m_* was determined with a saturating pulse of 8000 mmol m^-2^ s^-1^ PPFD with a 1-2 s of duration. The setting was as follows: act1 (%) = 30 %; act2 (%) = 30 %; super (%) = 10 %; shutter = 1; sensitivity (%) = 60 %. The maximum quantum efficiency of PSII photochemistry (Fv/Fm) was calculated using the formula Fv/Fm = (*F_m_* - *F_0_*)/*F_m_*.

**Quantitative analysis**

Arabidopsis images under UV light were split into red, green, and blue channels using Fiji([Schindelin et al., 2012](#_ENREF_7)). The total leaf area and the virus-infected area were was determined by the red channel pixels and green channel pixels, respectively, in the Analyze Particles function with size 100-infinity and circularity 0.0-1.0. The ROI manager function was used to produce the pseudocolor image.

**Statistical analysis**

All statistical analyses were performed by two-tailed Student’s *t*-test with Office Excel 2016 or GraphPad Prism 8.0.2. All data are represented as the mean ± SD. Source data are provided as a Source Data file.

**Data availability**

All data are available within the Article and Supplementary Files. All constructs and transgenic plants are available upon request.

Supplemental Figure legends

**Supplemental Fig. 1**

Confocal microphotos of *N. benthamiana* epidermal cells expressing proPep1^NIa-Pro^-YFP alone or together with NIa-Pro after plasmolysis with 30% sucrose. The cell membrane was labeled by mRFP-tagged phosphatase 2A catalytic subunit (PIP2A-mRFP); Scare bar, 10 μm.

**Supplemental Fig. 2**

**a**, Phenotypes of three-week-old seedlings of WT, *35S::proPep1^NIa-Pro^-YFP-1*, and *35S::proPep1^NIa-Pro^-YFP-3* under steady-state conditions. Scare bar, 1 cm. **b**, Western blotting for the expression of proPep1^NIa-Pro^-YFP in transgenic plants using polyclonal antibodies against GFP. PPS, Ponceau S staining.

**Supplemental Fig. 3**

**a**, Bar graph showing the ratio of TuMV-infected to total leaf area of WT and transgenic plants at 11 dpi (n = 3). **b**, Western blotting for the cleavage of proPep1^NIa-Pro^-YFP in transgenic plant by TuMV-6K2mCherry. **c**, Bar graph of the relative viral genomic RNA in WT and transgenic plants at 11 dpi (n = 3). *EF1α* was used as the internal control in the RT‒qPCR, and the genomic RNA in virus-infected WT was normalized to 1. **d**, Bar graph of the relative expression of *PR1* in WT and transgenic plants at 11 dpi (n = 3). *EF1α* was used as the internal control in the RT‒qPCR, and the expression of *PR1* in virus-infected WT was normalized to 1. * and ** indicate *p* <0.05 and 0.001, respectively, in the student’s *T* test.

**Supplemental Fig. 4**

**a**, Phenotype of WT and transgenic plants mechanically inoculated by buffer (mock) or TuMV-6K2mCherry under white and UV light at 11 dpi. Red and yellow colors in the pseudocolour panel refer to virus-free and -infected areas, respectively. **b**, Bar graph showing the ratio of TuMV-infected to total leaf area of WT and transgenic plants at 11 dpi (n = 3). ** indicate *p* < 0.001 in the student’s *T* test.

**Supplemental Fig. 5**

**a**, Phenotype of WT and transgenic plants agroinoculated by buffer (mock) or BSCTV at 14 dpi. The curly flower caused by TuMV are indicated by read arrows. **b**, Bar graph of the viral genomic DNA in WT and transgenic plants at 14 dpi (n = 3). *Actin II* was used as the internal control in the PCR, and the BSCTV genomic DNA in virus-infected WT was normalized to 1. **c and d**, Bar graphs of the relative expression of *PR1* (c) and *PR5* (d) in WT and transgenic plants at 14 dpi (n = 3). *EF1α* was used as the internal control in the RT‒qPCR, and the expression of *PR1* or *PR5* in BSCTV-infected WT was normalized to 1. n.s. indicates not significantly different in the student’s *T* test.

**Supplemental Fig. 6**

**a**, Western blotting for the cleavage of transiently expressed proPep1^AvrPphB^-YFP by AvrPphB-mRFP or AvrPphB^C98A^-mRFP in *N. benthamiana* leaves at 2 dpi. The black arrowhead indicates the cleavage product. **b**, Phenotypes of three-week-old seedlings of WT and *rps5 35S::proPep1^AvrPphB^-YFP* under steady-state conditions. **c**, Bar graph of the relative amount of *pst* DC3000 in *rps5* and *rps5 35S::proPep^AvrPphB^-YFP* at 40 hpi. *** indicate *p* < 0.0001 in the student’s *T* test.

Reference

Bent A, 2006. *Arabidopsis thaliana* floral dip transformation method. In: Wang K, ed. *Agrobacterium Protocols.* Humana Press, 87-104. (Methods in Molecular Biology; vol. 343.)

Cheng X, Lang I, Adeniji OS, Griffing L, 2017. Plasmolysis-deplasmolysis causes changes in endoplasmic reticulum form, movement, flow, and cytoskeletal association. *J Exp Bot* **68**, 4075-87.

Earley KW, Haag JR, Pontes O*, et al.*, 2006. Gateway-compatible vectors for plant functional genomics and proteomics. *Plant J* **45**, 616-29.

Lellis AD, Kasschau KD, Whitham SA, Carrington JC, 2002. Loss-of-susceptibility mutants of Arabidopsis thaliana reveal an essential role for eIF(iso)4E during potyvirus infection. *Current Biology* **12**, 1046-51.

Li Y, Liu X, Deng W*, et al.*, 2022. Fine mapping the soybean mosaic virus resistance gene in soybean cultivar Heinong 84 and development of CAPS markers for rapid identification. *Viruses* **14**, 2533.

Nakagawa T, Kurose T, Hino T*, et al.*, 2007. Development of series of gateway binary vectors, pGWBs, for realizing efficient construction of fusion genes for plant transformation. *J Biosci Bioeng* **104**, 34-41.

Schindelin J, Arganda-Carreras I, Frise E*, et al.*, 2012. Fiji: an open-source platform for biological-image analysis. *Nature Methods* **9**, 676-82.

Zhu H, Hu F, Wang R*, et al.*, 2011. *Arabidopsis* argonaute10 specifically sequesters miR166/165 to regulate shoot apical meristem development. *Cell* **145**, 242-56.

**Supplemental Table 1.** Primers used in the research

| Primer Names | Sequence (5’-3’) |
| --- | --- |
| proPep1-F | CAAAAAAGCAGGCTTCATGGAGAAATCAGATAG |
| proPep1-R | GCTGGGTCCCGACGCTAATTATGTTGGCC |
| Pep-Pro-R | CGTTGTGTATCACCAGGCCACAAAG |
| Pep-Pro-F | GGCCTGGTGATACACAACGACCTCCTCC |
| 207Pro-F | GTACAAAAAAGCAGGCTTCATGAGTAACTCCATGTTCAGAGG |
| 207Pro-R | GTACAAGAAAGCTGGGTCCCGACGTTGTGCGTAGACTGCCGTGC |
| NIa-Prom-F | CACTAAAGACGGCCAAGCAGGAAGTCCAATGGTGAGCACG |
| NIa-Prom-R | CCTGCTTGGCCGTCTTTAGTGCTAATCCAGTG |
| 207-F | CGTCGGGACCCAGCTTTCTTG |
| 207-R | CATGAAGCCTGCTTTTTTGTAC |
| Avr site-R | CTTTGCCTTGACCTTTGTGGCTTTATCCCCC |
| Avr site-F | CGTTGTGGGGGATAAAGCCACAAAGG |
| AvrPpHB-F | GTACAAAAAAGCAGGCTTCATGGCATCCTCTTCAG |
| AvrPpHB-R | CAAGAAAGCTGGGTCCCGACGCGAAACTCTAAACTC |
| AvrPpHBM-F | CAATATTTCTGCCGGCCTCTCCACGGAG |
| AvrPpHBM-R | GGCCGGCAGAAATATTGTTATGATTAAC |
| TuMV CP-RTF | CAGGTTTGACAGACGAGCAA |
| TuMV CP-RTR | CCAGAGGTTCCAGCGTTTAC |
| AtEF1α-F | TCACATCAACATTGTGGTCATTGGC |
| AtEF1α-R | TTGATCTGGTCAAGAGCCTCAAG |
